# Deported, homeless, and into the canal: Environmental structural violence in the binational Tijuana River

**DOI:** 10.1016/j.socscimed.2022.115044

**Published:** 2022-05-18

**Authors:** Alhelí Calderón-Villarreal, Brendan Terry, Joseph Friedman, Sara Alejandra González-Olachea, Alfonso Chavez, Margarita Díaz López, Lilia Pacheco Bufanda, Carlos Martinez, Stephanie Elizabeth Medina Ponce, Rebeca Cázares-Adame, Paola Fernanda Rochin Bochm, Georgia Kayser, Steffanie A. Strathdee, Gabriela Muñoz Meléndez, Seth M. Holmes, Ietza Bojorquez, Marc Los Huertos, Philippe Bourgois

**Affiliations:** a Department of Family and Preventive Medicine, University of California, San Diego (UCSD), San Diego, CA, USA; b Graduate School of Public Health, San Diego State University (SDSU), San Diego, CA, USA; c Pomona College, Claremont, CA, USA; d Epigenetics Programme, Babraham Institute, Cambridge, UK; e University of California, Los Angeles (UCLA), Los Angeles, CA, USA; f Proyecto Fronterizo de Educación Ambiental A. C. (PFEA), Tijuana, Mexico; g Prevencasa A. C., Tijuana, Mexico; h University of California, Berkeley (UC Berkeley), Berkeley, CA, USA; i University of California, San Francisco (UCSF), San Francisco, USA; j El Colegio de La Frontera Norte (El COLEF), Tijuana, Mexico; k Herbert Wertheim School of Public Health and Human Longevity Science, UCSD, San Diego, CA, USA; l Division of Infectious Diseases and Global Public Health, Department of Medicine, UCSD, San Diego, CA, USA; m University of Southern California, USA

**Keywords:** Environmental injustice, Water quality analysis, WASH access, Ethnography, Epidemiology, US-Mexico border, Police violence, PWID

## Abstract

**Introduction:**

The US deports more Mexicans to Tijuana than any other borderland city. Returning involuntarily as members of a stigmatized underclass, many find themselves homeless and de-facto stateless. Subject to routinized police victimization, many take refuge in the Tijuana River Canal (*El Bordo*). Previous reports suggest Tijuana River water may be contaminated but prior studies have not accessed the health effects or contamination of the water closest to the river residents.

**Methods:**

A binational, transdisciplinary team undertook a socio-environmental, mixed methods assessment to simultaneously characterize Tijuana River water quality with chemical testing, assess the frequency of *El Bordo* residents’ water-related diseases, and trace water contacts with epidemiological survey methods (n = 85 adults, 18+) in 2019, and ethnographic methods in 2019–2021. Our analysis brings the *structural violence* framework into conversation with an *environmental injustice* perspective to documented how social forces drive poor health outcomes enacted through the environment.

**Results:**

The Tijuana River water most proximate to its human inhabitants fails numerous water-quality standards, posing acute health risks. *Escherichia coli* values were ∼40,000 times the Mexican regulatory standard for directly contacted water. Skin infections (47%), dehydration (40%) and diarrhea (28%) were commonly reported among *El Bordo* residents. Residents are aware the water is contaminated and strive to minimize harm to their health by differentially using local water sources. Their numerous survival constraints, however, are exacerbated by routine police violence which propels residents and other people who inject drugs into involuntary contact with contaminated water.

**Discussion:**

Human rights to drinking water, sanitation and hygiene are routinely violated among *El Bordo* in-habitants. This is exacerbated by violent policing practices that force unhoused deportees to seek refuge in waterways, and drive water contacts. Furthermore, US-Mexico ‘free-trade’ agreements drive rapid growth in Tijuana, restrict Mexican environmental regulation enforcement, and drive underinvestment in sewage systems and infrastructure.

## 1. Introduction

[Fieldnote excerpt, July 2019]:

The chemistry team is taking water samples from the Tijuana River with glass bottles, handheld computers and probes. We are standing on the concrete canal, in the hot Tijuana summer sun, next to two SUVs we are using for fieldwork and are accompanied today by a documentary film-maker. The river water smells of feces—though we are all used to it at this point. There is trash and random debris scattered around by recent rainwater flooding. We are ~ 100 m from the Mexico-US border crossing, and we can see a few hundred people lined up on the bridge, waiting to cross into the US. Suddenly 3 or 4 residents of El Bordo come running frantically towards us on the other side of the river. I recognize one woman as a resident of the nearby compuerta (a large, partially open metal door covering a drainage tunnel that connects the city storm drain system to the river canal). A police officer is chasing her, but she ducks straight into her compuerta, and the officer stops in his tracks. He is apparently not willing to enter the pitch-black, several hundred-meter-long tunnel full of knee-deep of stagnant rancid water. Moments later, four police vehicles screech through the canal, chasing another half dozen worn-down looking river residents. Officers hop off the back of the armored pickup trucks, and start picking them off one by one, handcuffing them and pulling them into their vehicles. One of the river residents splits from the group and plunges into the river, soaking himself up to his waist. The officers are clearly unwilling to enter the water to catch him. They stop and just watch shouting into their walkie talkies. He walks to the far side of the water flow, and walks slowly now towards us, smiling and waving. Suddenly another police pickup truck screeches to a stop behind us, loudspeaker blaring, “Estas rodeado, sientate alli” [we have you surrounded, sit down where you are]. The river resident looks around and sits down on the ground [beside the water], giving up exhausted, evidently hoping to reduce chance of an on-the-spot beating in retaliation for having “resisted arrest”. An officer roughly handcuffs him and pulls him towards the paddy wagon. “Wow I can’t believe they didn’t beat him for running” says one of the NGO workers, “it must be because we are filming. I feel bad for that guy when they get him back to the station.” For the next hour or two, police cars, pickup trucks, vans, and motorcycles whiz through the canal rounding up residents randomly. We see half a dozen more arrests. We also see a few people escape successfully, however, by running through the river water. Later, another police cruiser arrives with a single officer driving. He goes systematically through the entrance to each compuerta, picking through the belongings left behind in flight, selecting objects with resale value from each entrance pile. He takes special interest in residents’ bicycles loading a few into his police vehicle. After it’s full, he wedges two more bicycles between the large metal bumper and the hood of his vehicle. He uses handcuffs to secure the unsteady extra bikes to his front bumper and speeds off. During the whole ordeal, the police ignore our presence, and cameras. They say nothing to us, and we do not dare engage them…

This *operativo* (police raid) is merely one of the many routine survival challenges faced by the residents of the neighborhood of *El Bordo*, inside and alongside the concrete canal that contains the binational Tijuana River as it weaves through the heart of Tijuana, Mexico. Typical post-raid challenges for those living or frequenting in *El Bordo* consist of: 1) evading capture in an immediate follow-up raid, 2) rebuilding shelters burned by police, 3) finding a safe and quiet place in the canal to defecate, 4) avoiding violence from nearby drug sellers and other residents, and 5) scraping together money for food, drinking water, survival items stolen/burned by police and for many, heroin and/or methamphetamine, that are sometimes sold or shared inside *compuertas* and temporary shelter encampments, to stave off crippling withdrawal symptoms. In this context, the Tijuana River offers both limited temporary protection and chronic peril. The flow of water in the center of the canal physically divides the larger concrete space into two halves. It offers a form of escape from routine police violence, as officers are often unwilling to foul themselves by fording it. It also creates numerous health risks, as exposure to the contaminated water is quasi-unavoidable for residents, especially when pursued by law enforcement (Roberts, 2017). Although the canal environment defines the space within which many experiencing homelessness and many deported migrants reside, its impact on their health outcomes and the centrality of police violence/corruption, the negative environmental effects have not been well characterized.

The United State (US)-Mexico border—and its related carceral institutions—has been identified as a nexus of *structural violence*, lying at the intersection of historical and political frameworks that drive high levels of health harms for migrants traveling to, and return from, the US (Dubal et al., 2021). Tijuana receives the largest number (20%) of returning Mexicans from the US (Unidad de Política Migratoria, Registro e Identidad de Personas et al., 2020), many of whom were deported and experience extreme vulnerability upon arrival, including police victimization, homelessness, and injection drug use (Contreras Velasco, 2016; Velasco and Coubes, 2013). Deportees and unhoused people frequently become victims of systemic discrimination, social isolation, stigma, and police victimization (Albicker et al., 2016; DeMyers et al., 2017; Neves-Silva et al., 2018; Pinedo et al., 2015; Welsh and Abdel-Samad, 2018). Individuals are often separated from their families and places of origin, and become de-facto stateless (Dreby, 2015). Many reside in Tijuana’s liminal urban spaces, and waterways (Flanigan and Welsh, 2020), especially *El Bordo*. In 2013, researchers from *El Colegio de la Frontera Norte* estimated that between 700 and 1000 people were living in precarious self-made shelters in *El Bordo* (Velasco and Albicker, 2013). Since 2015, Tijuana’s local law enforcement has cyclically removed people residing in *El Bordo* by force (Sánchez, 2019 ). Despite this, many return to reside in the canal zone at the expense of basic human rights to essential public infrastructures especially shelter, water sources, sewage disposal, and physical security.

The Tijuana River watershed covers more than 4400 km^2^ binationally between Mexico and the US (Gersberg et al., 2004; Gladstone et al., 2021; Rio Arronte et al., 2020). Its urban portion is almost entirely contained within the municipality of Tijuana. Here, the Tijuana River receives untreated and treated wastewater from three secondary wastewater treatment plants, along with untreated urban rainwater and irregular industrial, food-processing, and household runoff (Ayad et al., 2020; International Boundary and Water Commission United States and Mexico, 2017; US EPA, 2021). During the last 30 years, the city of Tijuana has grown quickly, flooded with economic migrants from southern Mexico and elsewhere in Latin America, and from returning deportees from the US. Tijuana’s many large US-owned factories represent a much-needed employment source in a wider neoliberal context within which US low-cost corporate industrial agricultural imports have bankrupted the rural small-holder *ejido* village-based agricultural economy. This rapid growth has strained the capacity of the wastewater processing system (California Water Boards, 2018).

Despite sewage services increasing by ∼1.7% every year, Tijuana cannot meet the rapidly rising social and environmental needs of the binational region’s political economic growth model (Navarro-Chaparro et al., 2016). Industrial waste and irregular urban settlements [without basic services such a wastewater and stormwater drainage systems] visibly pollute the river (Wakida Kusunoki and Piñón Colín, 2013). Approximately 21% of the city’s wastewater discharges untreated into the environment [i.e. river and canyons] (International Boundary and Water Commission United States and Mexico, 2017; Navarro-Chaparro et al., 2016). Available reports on water quality of stormwater runoff in Tijuana and of the Alamar River, the principal urban Tijuana River tributary, document levels exceeding Mexican and the US Environmental Protection Agency (USEPA) water quality standards for fecal indicator bacteria, organic matter, and nitrogen and phosphorus, suggesting acute health risk upon contact with water likely contaminated by sewage and industrial or agricultural production/food and meat processing discharges (Ganster, 2005; Health and Ecological Criteria Division, Office of Science and Technology, United States Environmental Protection Agency, 2012; Wakida and Riveles, 1997; Wakida et al., 2014; Wakida Kusunoki and Piñón Colín, 2013). These studies suggest that contaminated water could represent an important health risk (Colford et al., 2007) for residents of *El Bordo*. However, their data are between 10 and 30 years old and therefore may not reflect current pollution levels. Additionally, no prior study has specifically measured the water proximate to *El Bordo*’s largest settlements or distinguished between different water sources used by residents for different purposes. We propose that these details are critical to understanding the role of environmental pollution in shaping the risk environment for deported migrants, people who inject drugs (PWID) and displaced rural economy new immigrants in *El Bordo*.

In this article, we explore the role of the built environment in potentiating the unique health risks faced by this extremely vulnerable population. We conceptualize the health risks faced by *El Bordo* residents leveraging the frameworks of *structural violence*, and *environmental injustice*, determined in a binational context of immigration and drug policy, neoliberalism, and police brutality. The *structural violence* framework describes how social structures and inert political economic forces (e.g., global North/South inequality at the US/Mexico border) drive systematic harm though mechanisms of economic, political, legal, and social oppression (Farmer, 2004; Mehta, 2016; Rhodes et al., 2012). Many of the structural forces driving poor outcomes in this population—problematic drug use, HIV risk, police victimization, undocumented civil status and mental health problems—have been well characterized (Baker et al., 2021; Contreras Velasco, 2016; Goldenberg et al., 2011; Martinez et al., 2022; Pinedo et al., 2015; Sabo et al., 2014; Velasco and Coubes, 2013). The *Environmental injustice* framework describes when population are “deprived of the clean air and water necessary to perform” daily life activities such as eating, sleeping, and working (Alvarez and Evans, 2021; Bonds and Martin, 2016), in a disproportionate fashion for socially-, politically-, and economically-marginalized groups (Legarda and Buendía, 2011).

*Structural violence* is very broadly helpful for understanding the social, economic, and political forces driving high rates of morbidity and mortality for this population. However, we propose a further extension of *‘environmental structural violence’* to highlight the role of *environmental injustice*, including contamination and control of space, as a unique nucleus by which *structural violence* is enacted. Water is central to human existence, and the Tijuana River shapes the physical environment for the population we describe here. Methodologically, we assess the topic of interest in a transdisciplinary fashion (Messac et al., 2013), simultaneously characterizing the water quality of the Tijuana River with methods from chemistry, assessing the frequency of river residents’ water-related diseases and tracing their water contacts with epidemiological survey methods, and leveraging ethnographic methods to assess how social forces leading to poor health are enacted through environmental contamination and the control of urban space.

## 2. Methods

Our binational, transdisciplinary team of physicians, ethnographers, epidemiologists, and chemists undertook a socio-environmental, mixed methods assessment of water quality of the Tijuana River and the health and wellbeing of its inhabitants. We integrated chemical testing of water quality at the most densely populated region of the Tijuana River canal during the 2019 summer months, with semi-structured interviews, quantitative questionnaires describing demographic characteristics, water use and contact practices, health status, and behaviors, and several years (2019–2021) of classic anthropological participant-observation ethnographic fieldwork.

**Fig. 1.**
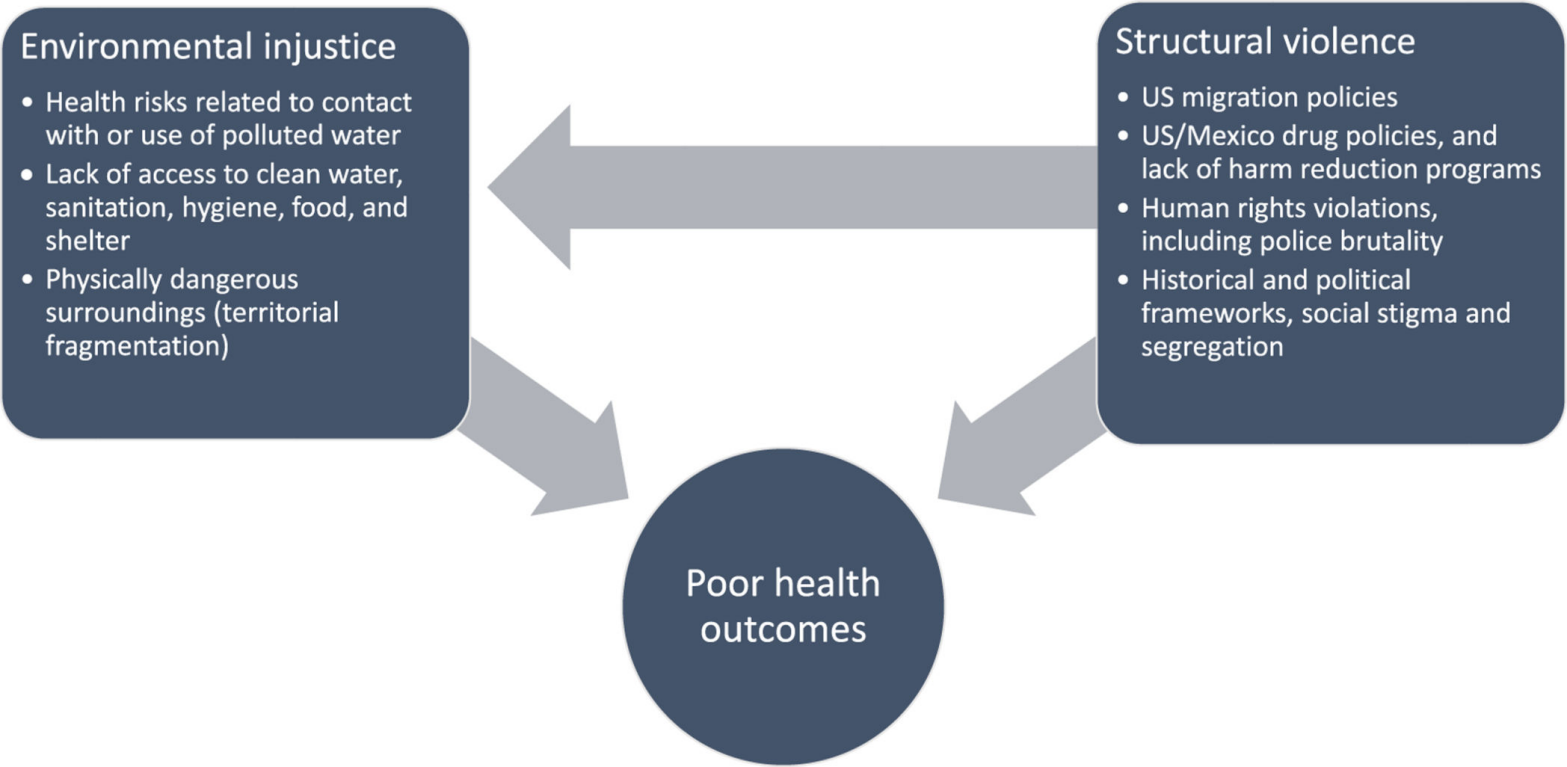
Environmental structural violence diagram. The *environmental structural violence* framework highlight the role of *environmental injustice*, as a unique nucleus by which *structural violence* is enacted to produce poor health outcomes.

By grounding our analysis of *structural violence* within an *environmental injustice* framework, we aim to address both the social and environmental factors that act in a synergistic fashion, imposing structural harms on the inhabitants of the Tijuana River Canal. In combination, both concepts identified as *environmental structural violence* represents the environmental and social conditions that drive poor health outcomes among the marginalized population described in this article (Fig. 1).

This project underwent institutional ethics review at [removed for blinded peer reviewed], in the United States, and [removed for blinded peer reviewed] in Baja California, Mexico.

### 2.1. Ethnographic methods

Numerous members of the ethnographic team [ACV, BT, SAGO, JF, PB, MD, ICA, SH, and CM] were trained in ethnographic methods and recorded ethnographic field notes before, during and following field outings over a several month period, as well as independently (2019–2021). Semi-structured interviews (n = 14) and ethnographic fieldwork notes (n = 22) in English and Spanish were collected inside the river canal during the water quality sampling in 2019. Adults (18+) who sought services from a harm reduction mobile clinic inside the Tijuana River Canal were the main informants. As chemical results were collected and processed, ethnographic and qualitative findings were brought into conversation and new rounds of data collection were planned. Longitudinal ethnographic data was also collected over 30+ months during 2019–2021 in Tijuana, Mexico by JF, ACV, PB, SH, and CM as part of wider studies examining shifting risk environments of PWID and urban border ecologies & environmental health. The environment of *El Bordo* was a frequent topic of conversation, both when conducting interviews physically in the canal and in other parts of the city. Longer-term fieldwork was targeted initially towards harm reduction venues, including a harm reduction mobile clinic inside *El Bordo*. It then organically extended to drug consumption and sales sites, residences, homeless encampments, and other street-based income-generation environments. Ethnographers accompanied PWID in routine daily activities. This relaxed interview style allows for more comfortably exploring a broad range of topics and enables documentation of risky and stigmatized practices [e.g., injecting, disputes among residents, scavenging] in real time as they occur. This approach to ethnography allows for accessing ‘common-sense’ understandings of topics related to homelessness, drug use, and policing, which can otherwise be difficult to assess reliably from routine survey instruments. Urgent strategies—e.g. those employed to evade police violence or quickly obtain resources to ‘get well [obtain drugs to prevent withdrawal symptoms]’—are routinely discussed by PWID in their daily scramble but this rich complexly nuanced data can be difficult to confirm in formal questionnaires. Long-term relationship-building and direct observation also reduces desirability biases, given that researchers associated with public health and medical NGOs can often be perceived as unrealistically *hypersanitary* or out-of-touch (Bourgois, 1998; Bourgois and Schonberg, 2009; Friedman et al., 2019). With IRB approvals, we undertook interviews in conversational and informal formats, frequently audio and/or video-recording them with participant consent. Targeted topical interviews were also conducted with strategically selected key informants (e.g. physicians, street outreach workers, emergency medical technicians, retail-level drug sellers, harm reduction practitioners, emergency medical technicians, law enforcement officers, and substance use treatment center employees) with especially relevant services and law enforcement institutional systems knowledge.

The final ethnographic database consisted of nearly 100 transcribed interview recordings, 500+ pages of fieldnotes, thousands of photographs, and dozens of videos. Data were entered into NVivo and ATLAS. ti, and analyzed for emergent themes using a grounded theory and structural vulnerability/violence approach.

During qualitative analysis, we selected representative quotes illustrating consensus or common occurrences relevant to public health human survival needs, service provision, violence, and law enforcement contact. To increase confidentiality, we use pseudonyms, and sometimes alter unimportant demographic information or logistics. Interviews were conducted in Spanish, English, and “Spanglish”, depending on informant preference. Transcriptions were translated by the ethnographers/authors to English with original Spanish or “Spanglish” terms in parentheses when relevant.

### 2.2. Survey methods

In order to generate quantitative data [for triangulation with ethnographic findings], the team of investigators [ACV, BT, SAGO, JF, ICA] administered questionnaires (n = 97) to n = 85 individuals during the summer of 2019. Questions were informed by initial ethnographic results, demographic characteristics and water-related health conditions and practices. Demographic questions included gender, place of birth, time living or entering *El Bordo*, migration to the US and deportation status, indigenous background, household and employment status, and details of intravenous drug use. Water-related questions included use/contact with Tijuana River Canal water (past week), and self-reported water-related health conditions.

Eligibility criteria included adults (18+) men and women recruited in the Tijuana River Canal in 2019 who consented to participate in the study, and who participated with [blinded for peer review] harm reduction mobile clinic interventions. Interviews were conducted in English or Spanish. Data were summarized numerically and graphically using R 4.0.3.

### 2.3. Water quality methods

**Fig. 2.**
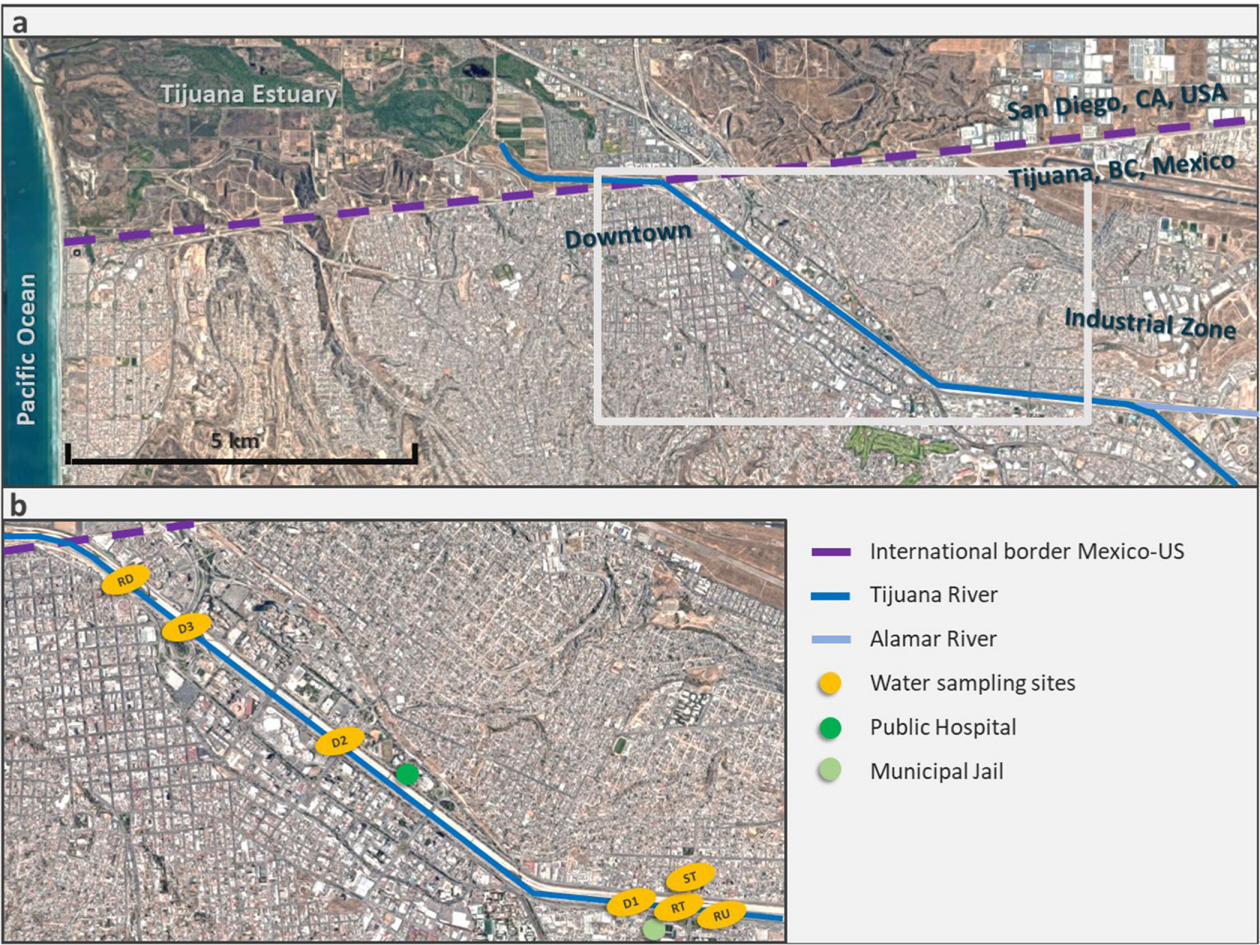
Tijuana-San Diego Border Region. **a.** Tijuana-San Diego Border Region. **b.** Tijuana River map and water quality sampling sites: river upstream (RU), river downstream (RD), storm drain (D1–3), riverside tube, *lloradero* (RT), and store tap (ST).

Surface water samples were collected from n = 7 sites in the Tijuana River canal during five sampling campaigns in June, July and August 2019 (Fig. 2). Two water sampling sites were from the Tijuana River directly upstream and downstream of the *El Bordo* population (sites RU and RD), three sites were active flows from storm drains into the canal (sites D1–3), one site was a riverside pressure-release tube – *lloradero* – (site RT), and one site was an outdoor tap of a convenience store (site ST). The latter two sites were selected due to ethnographic data indicating that they were frequently used by the study population.

We measured biophysical parameters, levels of fecal indicator bacteria [Fecal Indicator Bacteria - FIB; coliforms and *Escherichia coli* – *E. coli*] and chemical compounds found in wastewater. At the river sites RU and RD, water depth and turbidity [60 and 120 cm Transparent Turbidity Tube with Secchi Desk] were measured. Water temperature, pH, total dissolved solids (TDS), and dissolved oxygen (DO) were measured at all sites using a multiparameter sonde [Eureka Manta 2, Austin, TX] calibrated before each sampling campaign. All samples for bacteria and chemical testing were stored in sterile PET sample vessels or amber glass bottles rinsed beforehand with HCl and on-site with three volumes of sampling site water. Samples were stored at 4 °C until analysis. Total coliform and *E. Coli* [serial-diluted up to 10,000-fold] were enumerated using Colilert-18 kits within 4 h [IDEXX Laboratories, Westbrook, ME] for samples collected from sites RU, RD, RT, and SP. Samples were syringe-filtered within 8 h and diluted up to 50-fold prior to testing within 24 h by standard USEPA methods using Hach water quality tests. Samples for chemical analyses were collected at all sites and processed by USEPA compliant Hach methods 10,173 (Total Organic Carbon (TOC)), 8155 (nitrogen as ammonia), 8039 (nitrogen as nitrate), 8153 (nitrogen as nitrite), and 8048 (orthophosphate) within 24 h [Hach Company, Loveland, CO].

## 3. Results

### 3.1. The environment

[Fieldnote excerpt, August 2019]:

We are accompanying a mobile medical clinic offering medical services near one of the largest groups of tents and makeshift shelters in El Bordo. The clinic doctors are draining abscesses, handing out antibiotics and bandages, and administering HIV and Hep C tests. The harm reduction team is giving out sterile syringes and naloxone. A man using crutches makes his way slowly over the scavenged (and very rickety) metal highway barrier serving as a bridge connecting the two halves of the canal, which are separated by the quickly moving river water in the center. I am afraid he is going to lose his balance and fall into the filthy water, or that the bridge is going to give out as it bows under his weight, but he makes his way to our side uneventfully. He comes up to me, and begins telling me something, looking agitated. At first, he is difficult for me to understand because he is missing many of his teeth and speaks in a rapid-fire Spanish mumble. But quickly I come to understand that he is worried because his wife is on the other side of the canal, hidden behind the large concrete barrier of the bridge support, and she wants to see a doctor. She won’t be able to cross the metal bridge, however, because she is in a wheelchair. I pass along the message to one of the clinic doctors, and she assures the man that a few of the doctors will head over to the other side of the river once they finish up with everyone who is already in line to be seen. The harm reduction team decides to head over the bridge to hand out supplies in the encampment and assess how many people need medical attention. Just then, a couple of guys throw a huge coil of scavenged metal fencing over the nearby edge of the canal, and it bounces down the steep embankment, landing close to the water. They load it up into a shopping cart, and then, to my utter amazement, try to push the shopping cart—laden with what must be over 50 kg of metal—onto the tiny metal bridge. They miraculously get the cart up onto the beginning of the metal bridge, which starts to bend and dip into the river. They manage to push the cart a few meters further, before the bridge bends so much that it’s completely underwater, and the cart is totally stuck. I start to wonder how the harm reduction team will make it back over the river if the bridge is out of commission, but then both of the men, the cart, and the metal all fall tragically into the river with a big splash and bouts of laughter from all who observed the scene. The two men come up for air, rubbing river water out of their eyes, and laughingly mocking one another “por que lo dejaste caer, pendejo!” [why did you let it fall stupid!] Luckily, the water level was low, because it was a dry time of year, and no one was injured.

**Fig. 3.**
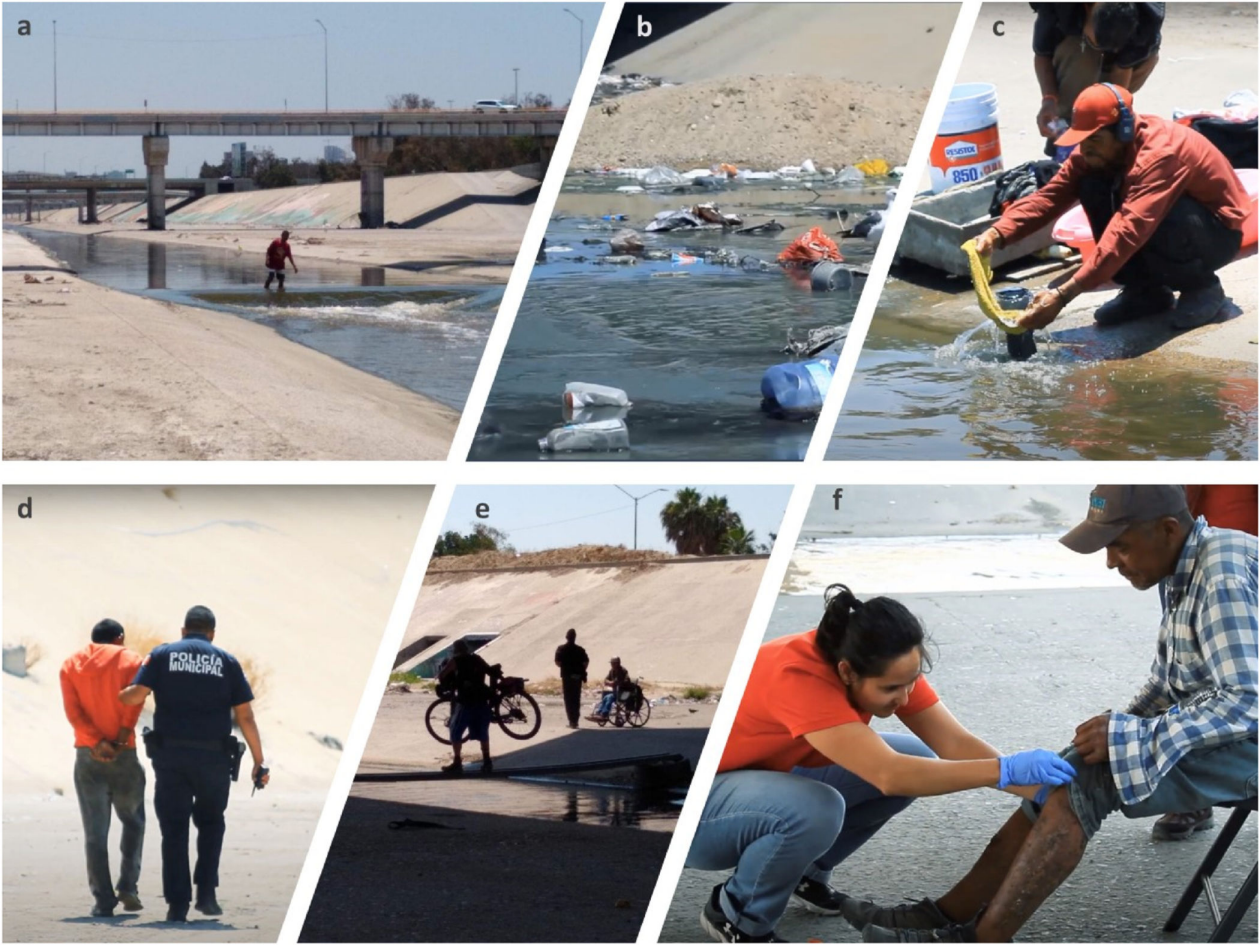
Images of *El Bordo.* **a.** A *El Bordo* resident crossing the Tijuana River canal by foot. **b.** Contaminated water flowing from a storm drain into the river. **c.** A *El Bordo* resident cleaning his belongings in *lloradero* water just above the river water. **d.** A police officer arresting a resident of *El Bordo*. **e.** A makeshift bridge used to cross the river. **f.** Mobile clinic service for PWID. Source: Pictures taken during fieldwork inside the Tijuana River Canal in 2019. Source: Satellite from Imagery

The river water running through the center of *El Bordo* physically shapes the space in which life can be conducted for its residents, serving as a key factor shaping the environmental health risk for its inhabitants (Rhodes et al., 2005). Its depth, and residents’ commonsensical aversion to contact with its contaminated, foul water, led to numerous logistical challenges and trade-offs. In a few places, precarious makeshift bridges have been put into place using discarded smashed metal guardrails from the highway sandwiching both sides of the cement sewage drainage basin. Only one person at a time can make the journey over these rickety structures, and falls are common. The fastest way to cross the river is to ford on foot. This requires becoming soaked—potentially up to the waist—in brown, pungent, water-soaking shoes, abscesses and cuts commonplace on the feet, legs and groins/genitals of canal residents (Fig. 3). But exactly for this reason, fording the visibly foul river, and facing its potential health consequences, is a primary tactic for escaping routine police violence. The officers are unwilling to risk touching the water themselves. In most instances exposure to filthy water is less risky to health than the prospect of a police beating, and carceral detainment for 36 h regardless of just legal cause (Morales et al., 2020) which is long enough for PWID consuming opioids to suffer excruciating *malilla* [withdrawal symptoms] (Syvertsen et al., 2010). Avoiding the river water, and avoiding police violence, represent competing risks to be navigated dynamically. The abusive effects of police harassment of marginalized and drug-using populations residing on the street is consistent with decades of research on law enforcement practices contradicting harm reduction priorities and dispossessing unhoused populations of possessions essential to basic survival needs (blankets, warm clothes, tents, makeshift shelters) (Bourgois and Schonberg, 2009; Syvertsen et al., 2013).

The Tijuana River canal is a wide, open urban space, easily accessible by foot and car from the nearby highways that line it on both sides. The canal bottom is ∼50 m wide, and the river runs in a smaller central channel that is approximately 5 m in width and lined with algae. The water has a green-brown color, has a strong odor of wastewater, and is highly turbid due to suspended organic matter and sediment. Trash and discarded objects have washed into many canal areas from upstream population centers. Often, the field team observed dead animals and—once—a human body. The canal is lined with gated storm sewer openings (*compuertas)* that discharge water from a citywide network of surface storm drains. Many of these openings serve as semi-permanent homes for inhabitants, who enjoy the protection afforded by the physical barriers and darkness, despite the constant risk of flooding and exposure to contaminated water. Some of these *compuertas* also serve as pop-up, informal sales points for injectable opioids and/or methamphetamine. Despite the arid climate, the Tijuana River experiences constant flow due to wastewater discharges in the urban environment. During Tijuana’s infrequent rainstorms the entire canal floods, washing away homes, crucial survival possessions and makeshift bridges.

At the bank of the canal’s central river channel, there are numerous −10 cm diameter, <15 cm tall plastic tubes called *lloraderos*, that actively trickle clear water onto the canal surface (Fig. 3). Depending on the level of the river flow, *lloraderos* can be up to 2 m away from the river water or completely submerged. The appearance of water from *lloraderos* seems cleaner than the Tijuana River flow; it is clear and without odors that could indicate pollution. Although *lloraderos* are visually inconspicuous and have been overlooked in the existing literature, we found that they were critically important water sources for residents of *El Bordo*.

**Fig. 4.**
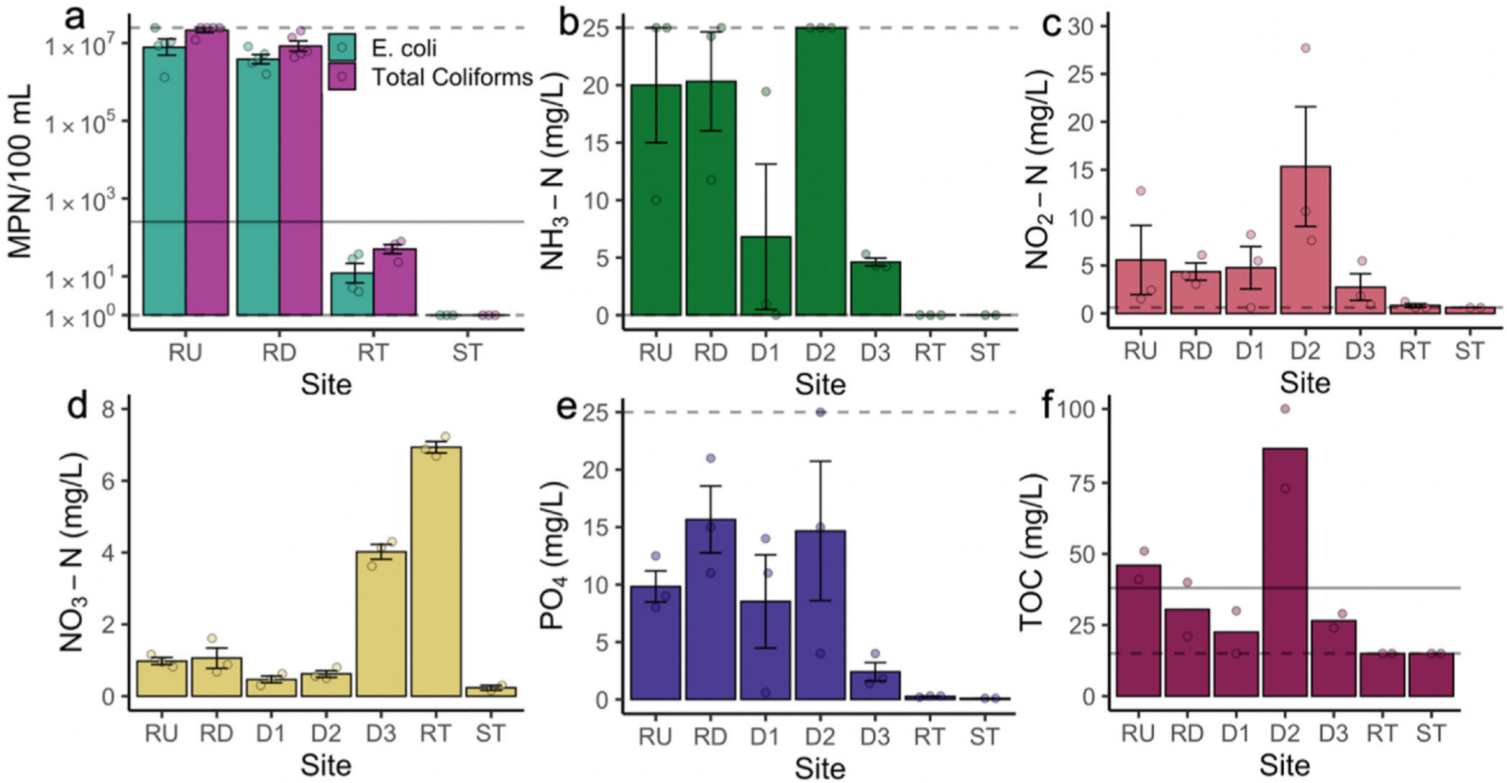
Levels of important biological and chemical water quality indicators in *El Bordo*. Gray dashed lines indicate detection thresholds (maxima or minima). Gray bar indicates the Mexican regulatory 30-day-mean water quality standard (NOM-003-SEMARNAT-2021). **a.** Total coliforms and *E. coli* at river (water quality standard is for *E. coli*) and frequent-use (f and h) sites. **b-d.** Nitrogen in ammonia, nitrites, and nitrates. **e.** Phosphate (orthophosphate). **f.** Total organic carbon (TOC).

Levels of fecal indicator bacteria [FIB; coliforms and *E. coli*] and chemical compounds commonly used to assess pollution levels and health risk of contact with wastewater are presented in Fig. 4. We found that water from the river and storm drain sites was slightly alkaline (pH 7–8) and possessed large amounts of total dissolved solids (1500–4200 ppm), consistent with a significant proportion of the flow being untreated sewage. Tijuana River *E. coli* levels were four orders of magnitude above [ –40,000 times] the Mexican legal limits for treated wastewater directly contacted by humans [*Norma Oficial Mexicana NOM-003-ECOL-1997*], and river water [*NOM-001-SEMARNAT-2021]* (Diario Oficial de la Federación, 1998 ; Secretaría de Medio Ambiente y Recursos Naturales, 2021). In effect, levels of FIB were similar to those found in untreated sewage (WHO, 2003). Total coliform levels were about three orders of magnitude greater than reported by the Mexican Federal Government for the Tijuana River in 2019 (CONAGUA, 2021), and one to two orders of magnitude higher than measurements in the Tijuana River between 1995 and 1997 (Wakida and Riveles, 1997). Detectable levels of *E. coli* in water from the riverside tube *lloraderos* indicated that it was not a safe source of drinking water, although contamination levels were far lower than those observed in the flowing river water. FIB could not be detected in tap water from the convenience store site. Total inorganic nitrogen [the sum of nitrogen in ammonia, nitrates, and nitrites], an indicator of microorganismal activity and animal waste, was above the Mexican legal limit of 25 mg/L for total nitrogen at river and drains sites RU, RD, and D2. It is important to note that total nitrogen and phosphorus levels were not measured and will be higher than total inorganic nitrogen and phosphate levels, so additional sites may exceed the nitrogen and phosphorus water quality standards. Moreover, ammonia and phosphate river water levels were an order of magnitude higher than levels measured in the Tijuana River between 1995 and 1997 (Wakida and Riveles, 1997). TOC, an indicator of microbial load, was above the Mexican legal limit of 38 mg/L for sites RU and D2 (Fig. 4).

Together, these data indicated significant contribution of raw sewage to overall poor water quality in the river and storm drain outflows, especially D2, where *E. coli* levels from a single sampling campaign exceeded the upper detection limit of the method. Comparing to prior literature, we find that the water quality of the Tijuana River has worsened over time. According to Mexican and International water quality standards, ingestion of riverside tube water (*lloraderos*) and skin or orifice contact with river or storm drain water would pose acute health risks to residents.

### 3.2. The residents of El Bordo

The population of the Tijuana River canal consists of a constantly changing – though often returning – set of residents who face extreme social marginalization. Survey participant characteristics (n = 85) are described in Table 1. The average age was 41-years-old (range 21–65 years), with an average educational attainment of 8 years (range 0–16 years). Most residents were male (92%), and it was common for no women to be seen in the canal during a day of fieldwork. Just one participant was self-identified as Indigenous (Yaqui). No children were observed in the main areas where dense clusters of makeshifts houses are located. In the main population centers, improvised shelters (*ñongos* ) were made with sofas, tarps, cardboard, mattresses, and other scavenged materials that keep residents warm year-round during cold, windy nights typical of the canal. The composition and number of *ñongos* changed quickly over time, especially as police and other government workers frequently burn these residences, and heavy rains sweep them away.

Almost all residents of *El Bordo* were born in Mexico, with the largest percentage from Baja California (42%), followed by southern (33%) and other northern Mexican states (20%). A small minority of participants were born in the US (2%), and no other countries were represented. Nevertheless, the majority of participants have lived in the US (61%), with a median of 13 years (range two months to 47 years) spent there. Most of who had lived in the US were deported (83%), on average 10.4 years before the time of the survey (SD 7.4).

*“It’s already been two years [since I was deported] … they just left me here to grow old, and there’s nothing I can do. When I was in California, I never thought it could come to this. I mean I come from a good family, we weren’t poor. We were middle class. Businesses, cars, we weren’t missing a thing. But I got caught with drugs and lost my green card, got deported, lost everything. Most of my siblings are [US] citizens and everything.”* – Javier, 59-year-old man, originally from Ciudad Juarez, Mexico, deported from California two years prior, living in *El Bordo* for the last six months.

The median time that participants have been living or spending time in *El Bordo* was 3.3 years (range 2 days–45 years). Most respondents lived in the river canal, although some had housing elsewhere (7%) and only enter the canal to purchase drugs and socialize. It was common to find individuals who typically resided in other parts of Tijuana spending short periods of time in *El Bordo*, as the canal is very proximate to the large municipal jail where marginalized people were frequently taken by police. After leaving jail, many stopovers in the canal quickly purchase or beg/borrow drugs, to ‘get well’ or rest temporarily free from police surveillance, before starting the journey/trek back to one’s home area of the city. Although violent conflicts occur within the canal, many informants also spoke positively of the community found there.

*“Yeah, I’ve only been sleeping here for a little while, but I like it. The people here are nice, and we take care of each other. I get the best sleep when I’m in the canal*—*with canal people*—*since I know the cops are way less likely to bother me here. When I sleep here, of course, they ask [for sex], but they’re asking you politely, and if you say, ‘don’t touch me’, they don’t. The first couple times coming here at night I was scared. I was like: ‘Dude, they could really mess with me’. But they’re all very respectful and people actually share things here and take care of each other.”* – Jessica, 38-year-old woman originally from Texas, US, has been living in *El Bordo* for the past two weeks.

Most residents of *El Bordo* do informal work such as scavenging, recycling cans, washing cars at stoplights, temporary assistance in swap meets and markets, and selling goods on the street. The long line of cars, waiting for several hours to enter the US, forms next to the canal and offers a captive audience willing to purchase food and other goods, tip for services like car cleaning, or giving money out of charitable gesture. Others work doing manual labor. Residents are flexible, adapting themselves to the opportunities that present themselves.

*“Well, sometimes I go and help them in the swap meet … to set up tents in the morning and break them down in the afternoon. I basically do whatever they tell me. If there is an opportunity to clear a field, or sweep someone’s store, help someone take care of their pet … whatever they want … if I’m going to end up with a little bit of cash … that’s what I do.”* – Pedro, 33-year-old man, originally from Sonora, Mexico, living in *El Bordo* for three years.

### 3.3. A taxonomy of water use and exposure

Although the residents of *El Bordo* lived near a major waterway, accessing improved water sources was difficult. *El Bordo* residents reported chronic difficulties accessing drinking water, sanitation and hygiene (WASH) [e.g. latrine, bathing, and handwashing facilities] near the canal. This impacts all residents’ health, including menstrual health among female residents [i.e. lack of privacy to manage menstruation]. *El Bordo* residents often preferred to access sanitation and water sources from local private businesses and public restrooms that require paying a fee.

*“When they’ll let me in, I wash my clothes at the gas station when I go into the restroom. Sometimes they’ll let me do it because I clean the restroom for them. That’s how I pay to use the bathroom, I have to clean it. But at least I get to wash my stuff. Plus, I can sometimes fill up a bucket with water from the tap and bring it back here with me”* – Max, 38-year-old man, originally from Guerrero, Mexico, work cleaning cars, deported from the US.

However, due to their stigmatized drug use, deportee status, and physical and/or economic limitations to accessing services at local businesses, open defecation was a common practice inside the river canal, occurring in partial privacy behind concrete barriers or in the darkness of evening hours.

In the popular press and even some academic articles describing the residents of *El Bordo,* it is commonplace to find the assumption that residents carelessly routinely sought untreated river water for consumption and preparation of drugs for injection (Werb, 2019). Nevertheless, our team never observed such a high-risk practice in many participant–observation and harm reduction service provision interactions. Participants emphatically denied that canal water was used for either consumption or injection:

*“Hell no! I have never seen anyone using the river canal water to drink, prepare drugs, or clean wounds … No, no, no. Because we all know that it can give you the chills, and really hurt you. You’d have to be really out of your mind. Really fucked up. Someone is usually going to help you out with a little bit of clean water. And even if not, worst case scenario we’d just use the lloradero”* – Bruno, 45-year-old man, originally from Michoacan, Mexico, plumber, deported from the US, who has resided in *El Bordo* for 20 years.

Water from *lloraderos* is considered a lower risk source but is still only used sparingly for delimited tasks such as washing feet, hands, clothes, dishes, and the yellow flannel rags that river residents characteristically use to clean cars for tips at stoplights and in border crossing line traffic jams. This is where many *El Bordo* residents generate their steadiest income legally in Mexico’s huge informal personal services and sales economy. *Lloraderos,* therefore, represent an important free and accessible water source for the canal residents.

*“No pasa nada con el hoyito [nothing bad happens with the water from the lloradero] … the water from that one over there comes out real clear and nice. It’s definitely not river water coming out of that hole, you can just tell, it’s gotta be water that seeps out from the ground and bubbles up.”* – Javier, 59-year-old man.

*“Yeah, I sometimes use the lloradero water. My feet were really hurting this week, since they were burned in a fire, so I took off my shoes and washed my feet with soap and the water that comes out of lloraderos. But just for stuff like that, I don’t use it to wash my face, or my hair, or to bathe, or to inject myself, or to eat …”* – José, 34-year-old man, originally from Tijuana, lived in California for 19 years, deported from the US 10 years ago, and living in *El Bordo* since then.

**Fig. 5.**
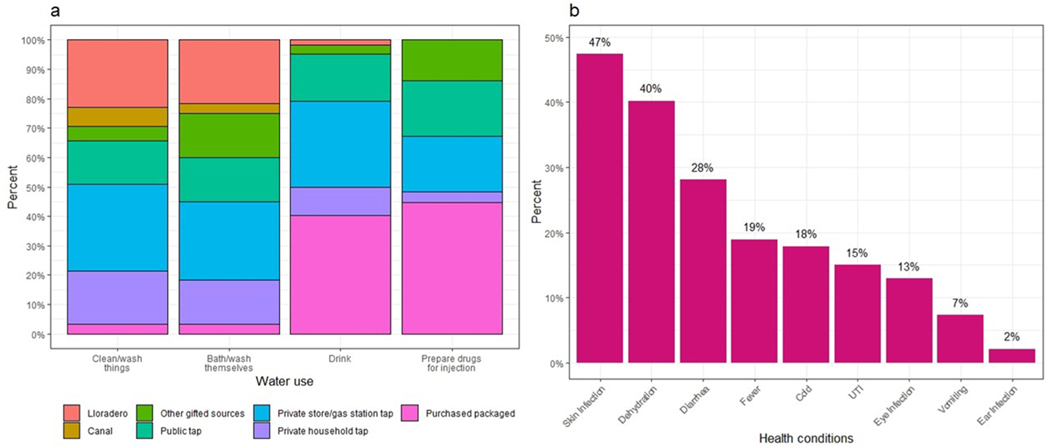
*El Bordo* residents’ interaction with water sources. **a.** Principal water sources in last week. **b.** Self-reported health water-related conditions. Source: 97 questionnaires administrated in 2019.

River residents have commonsensically developed a risk-reduction *taskonomy* (Dougherty and Keller, 1982; Nichter, 1993) and taxonomy of water sources and practices to minimize harms in the face of social marginalization and fear of police harassment (Nichter, 2006). Water sources were generally preferentially ranked as: 1) bottled or packaged water purchased from convenience stores or distributed free by harm reductionists or evangelical Christian missionaries and other humanitarian actors who periodically visit the canal and offer services (Panter-Brick, 2022), 2) tap water obtained from nearby taps outside of private businesses or houses, 3) *lloradero* water, ideally boiled over an open flame, and 4) water from the canal. This taxonomy aligns with the quantitative water quality results we documented above (Fig. 5). The tap water from sites highly frequented by residents of *El Bordo* tested potable, without evidence of contamination. *Lloradero* water did contain some bacterial contaminants, but at much lower levels and frequencies than untreated river water.

Survey results also confirmed that purchased, packaged water was the most common source to drink (40%) and for preparing drugs for injection (44%) (Fig. 5). Tap water from households, small business, gas stations, and saline solution provided by harm reduction services were also preferential sources. Reflecting the tradeoffs of cost, convenience, and perceived risk, water source varied strategically by type of activity. Private stores or gas station tap water was the most common source for cleaning or wash things (30%), and for bathing or washing oneself (27%), and it was a frequent source for drinking (29%) and for preparing drugs for injection (19%). Public taps such as from parks and sprinklers [municipal tap water], private household taps, and gifts of water were also commonly mentioned. *Lloradero* and Tijuana River canal water were used mainly to clean/wash things (23%, 7%), such as dishes and laundry, and to bathe/wash distinctly vulnerable parts of one’s body, e. g. face and hair versus hands and feet (22%, 3%).

Apart from these routine and intentional uses of water, the everyday emergencies of police raids and daily income generation and travel to encampments and sales sites logistics often force residents to involuntarily interact with contaminated river water. As described in the opening passage of this article, the most reported reason for immersion in canal water was to flee during regular police raids, which shifts in frequency from daily to monthly or weekly, depending on the vagaries of the local political climate. Most participants used, or were in contact with, Tijuana River canal or *lloradero* water – for any of these reasons – at least once in the previous week (73%). In the prior week, the most common uses and contact with the canal and the *lloradero* water sources were rinsing hands (63%), feet (52%), face (40%), and eyes/ears (35%), washing hands (53%) and face (35%), and doing laundry (38%) (Table 2). Some also used water to bathe (38%), wash dishes (30%), and rinse (22%) and clean wounds (19%). A minority used it to cook food (10%), rinse their mouth (7%), brush their teeth (7%), prepare drugs for injection (3%), or drinking (1%).

### 3.4. Health status and access to healthcare

The most common self-reported and potentially water-related health conditions were skin and soft tissue infections (47%) associated with river water use/contact [OR: 2.3, 95% CI: 1.2–5.3]. Many river residents were observed with large volume abscesses—referred to as *cuerazos*—that cause significant levels of pain and impair mobility (Fig. 3). Abscess formation was often attributed by river residents to injection practices and insufficient hygiene services, as well as lingering injuries from police beatings. One frequently observed condition was of chronic diarrheal discharge. Many specifically identified contact with river water as an exacerbating factor and described skin infection as subjectively proceeding after being submerged in the river. Symptoms including itching, burning, irritation, and skin hives were mentioned most frequently.

*“I used river water to bathe once. Then I got all these little bumps all over my skin and had to go see a doctor … like welts on my skin. I had to go get a cream at the hospital. I think it was definitely the water, the canal water.”* – Javier, 59-year-old man.

In survey data, many participants also reported thirst and hunger, dehydration (40%), diarrhea (28%), and fever (19%) in the week prior to the survey (Fig. 5). The residents attributed these symptoms to limited access to clean water and precarious quality and quantity of food and beverages.

*“I drank water from the lloradero that day … well because I was hungry and thirsty and didn’t have anything else … but it gave me a stomachache. To be honest, I am ashamed … but that night, I didn’t make it to the bathroom, I had to throw away my underwear.”* – Tenoch, 53-year-old man, originally from Guerrero, Mexico, lived in the US for 22 years, deported and has been living in *El Bordo* for two years.

Motor and sensory disabilities were common, especially amputations, and so were broken bones with related sequelae. A surprisingly large number of residents used wheelchairs and crutches in challenging broken terrain and despite risk of falling into the water when fording a canal. The most common causes of these injuries were vehicle-on-pedestrian accidents. The canal is lined by a 4-lane highway on each side, and river residents must traverse the highways on foot many times each day to access food, income, and basic goods and services. Combined with pre-existing mobility limitations, and the psychoactive influence of polydrug and alcohol use promotes accidents that are highly debilitating in this challenging environment. The high rate of catastrophic motor vehicle on pedestrian accidents were also a function of the local built environment. Despite *El Bordo* being home to large populations for decades, there was no safe pedestrian access to or through this large canal basin. Although pedestrian access points were added to the canal by a previous municipal administration, they were promptly removed. The need to cross a perilous stretch of highway to access *El Bordo* is just one more fashion whereby the structure of the city’s environment (territorial fragmentation) drives poor health outcomes for residents of the river zone.

Most study participants injected and/or smoked both heroin and methamphetamine (78%), some consumed only heroin (15%), or neither drug (7%). Some also smoke cannabis or tobacco.

In practice *El Bordo* residents, especially PWID, had extremely limited access to health care services. Many utilize harm reduction-oriented medical services from outreach organizations, but structural barriers prevent reliable access to hospital care. Although Mexican hospitals ostensibly offer universal healthcare to all citizens, numerous logistical and stigma-related barriers make accessing it very difficult. For instance, most river residents do not have identification to prove eligibility. Further, public hospitals generally do not provide treatment for opioid use disorder or prescribe opioids to patients for almost any reason, and so crippling withdrawal symptoms cause many PWID to leave the hospital early. In reality, most individuals who are perceived to ‘look homeless’ simply cannot get past the hospital’s door guard. Police contact often further complicates treatment, as officers throw-away needed medications and prevent participants from getting to HIV and tuberculosis clinics.

*“I have wanted to go [to the harm reduction medical clinic in the city center] but I honestly can’t get there most of the time because of the police. Before I can make it too far, they catch me. And even if I make it, then on the way back, they always catch me. They take one look at me and search me. And even if they don’t find anything, even if I don’t have any syringes* – *bam! I get 36 hours in jail just for being on the street.”* – Vincent, 32-year-old man, originally from Tijuana, never lived in the US, unemployed, has been living in *El Bordo* intermittently over the past four years.

*“I went to the [public] Hospital but I didn’t have my [ID] card … they threw me right out on the street, didn’t even want me in the building. Even though an ambulance took me there, they threw me out on the street. And I slept on the fucking sidewalk right there in front of the hospital that night.”* – Antonio, 50-year-old man, originally from Zacatecas, Mexico, lived in the US for 18 years, deported, and living in *El Bordo* for 15 years.

### 3.5. Vulnerability to abusive and violent policing

“The police force you in [to] the canal. And then, other times they’re coming in and forcing you out of the canal. And really you can’t be in or out, because they’ll beat you and take you to jail either way. They want you in jail all the time. They pretty much don’t want us to exist at all.”

– Antonio, 50-year-old man.

As described in the opening passage of this article, we observed several municipal police raids inside *El Bordo*. These were often referred to in local Tijuana political discussions as components of *limpiezas* [cleanings], where in theory, the canal was returned to its “natural state” of barren, infertile, uninhabited concrete. Large trucks collected trash that has washed into the canal, as well as mountains of dirt, and vegetation from the canal floor. Police rounded up the inhabitants, took them to municipal jail, and/or shipped them to far-off drug rehabilitation facilities against their will (Loera and Margarita, 2017; Rafful et al., 2020) (Fig. 3). Despite police raids, one of the most common reasons that canal residents reported choosing to live in *El Bordo* rather than on the street is that it represents an environment with relatively less risk of police violence and detention. Canal residents reported that they could go for up to a week without being beaten, extorted, or detained by the police. In contrast, in many other neighborhoods, unhoused people suffered inevitable daily punitive encounters with officers. The canal also offered better chances of escaping police capture, as individuals can flee into storm drains, or cross the river, in effect escaping one form of bodily harm by exposing themselves to another health risk.

Among unhoused individuals in Tijuana, there was a natural tradeoff between the ability to earn money and risk of police violence. Living and spending time closer to the border means an increased ability to gather funds, as it facilitates rapid access to the multiple hours long lines of cars waiting to cross into the US. It is also proximal to the entertainment, sex-and drug-tourism services provision markets within walking distance of the US border crossing. These sites offer diverse opportunities for informal sales of cheap merchandise, odd service jobs (car washing, cleaning local businesses and tips from tourists and drug sellers). However, for similar reasons, closer to the border, police attention was also much more intense and more hostile. The more distal residents of *El Bordo* often chose quieter, albeit less lucrative shelter residence environments, where money was harder to come by, but the environment less hyper-policed. The decision to live in the canal flags more extreme social marginalization, and increased health risks from contact with water and exposure to the toxic elements in the open canal environment (filthy water, abusive police raids, and pedestrian traffic accidents).

## 4. Discussion

The human right to potable, accessible, affordable, and sufficient water is frequently violated among unhoused people, damaging their quality of life, health, dignity and exacerbating social exclusion (Neves-Silva et al., 2018; Uddin et al., 2016). Few studies, however, have investigated homeless populations in their fuller daily experience of their infrastructure, seeking refuge in *de facto* semi-abandoned public spaces of rivers and sewage/rainwater drainage canals to avoid police harassment, find temporary partial privacy and fleeing public embarrassment (Flanigan and Welsh, 2020; Friedman et al., 2021; Pinillos, 2020; Verbyla et al., 2021). This lacuna is surprising because access to basic WASH is the most crucial major urban environmental infrastructure necessary for human survival, a core concern at the dawn of the emergence of the discipline of public health, and also fueled the development of epidemiology and urban planning into well-funded, policy--relevant disciplines (Rosenberg, 1966). Our ethnographic data and survey statistics demonstrate how negative health outcomes due to forcible contact with water are precipitated by law enforcement violence both proximally and distally. Possessions crucial to housing, quality of life and survival—including irreplaceable nationality identification cards necessary for accessing public hospital care (Pinillos, 2020)—were regularly stolen or destroyed by violently punitive police officers during raids in the name of the local government authorities. In this context, the semi-abandoned liminal public space of the polemically iconic US/Mexico liminal border wall is turned into a violent no-go-zone [see (Flanigan and Welsh, 2020; Palta et al., 2016) for comparative examples]. Our detailed social documentation of water use practices and laboratory analysis of water quality at distinct sources reveals how a public health, environmental analysis of the inadequacy of Tijuana’s sewage infrastructure benefits from an understanding of structural political and economic forces of local government management, Mexican law enforcement logics, and US migration and drug policies. The confluence of these factors drives disastrous health outcomes for deported migrants and dislocated rural/urban internal displaced people seeking urban jobs or striving to cross the US border (Fig. 1).

The failure of the state to provide WASH services is a key upstream infrastructural deficiency exceptionally damaging the health of unhoused and stigmatized vulnerable populations who use drugs. The highly marginalized population of deportees and unhoused rural/urban internal displaced has been rendered even more vulnerable to poor health outcomes through intersecting social [political, economic, cultural] and environmental infrastructural forces (Saxton, 2015). In Tijuana, as in other locations, unhoused populations are often not viewed as ‘members of the community’, but instead as pollution to be ‘cleaned’ (Bonds and Martin, 2016). As a product of US immigration and prohibitionist drug policies, many borderland residents have become quasi-stateless: they were deported from the US but either never possessed Mexican identification papers or lost them during abusive police raids. Arriving in Tijuana, deportees enter a socially hostile, physically insecure, contaminated environment, with limited economic opportunities and severed social supports. Brutal police target anyone “looking homeless” (Pinedo et al., 2015). Rates of hepatitis C and HIV are high among PWID deportees, access to healthcare is extremely limited, and mortality/morbidity health outcomes are shockingly high (Pinedo et al., 2014; Strathdee et al., 2008).

Our water quality testing documented high rates of contamination in river water. It fails numerous water-quality standards, particularly *E. coli,* TOC [an indicator of microbial load], and total inorganic nitrogen [an indicator of microorganismal activity, animal waste, and in industrial production run-off]. This demonstrates that the river water poses an acute health risk to anyone who contacts it and is contaminated by untreated sewage due to inadequate public wastewater management. Our data also suggest that FIB, ammonia and phosphate levels have significantly increased in the Tijuana River since the early years of the North American Free Trade Agreement (NAFTA) between Canada, Mexico, and the US (Wakida and Riveles, 1997), in a worsening trend. Future analyses of environmental impacts in Mexican border cities should focus on establishing longitudinal water quality monitoring programs through collaboration between universities, binational government agencies, and environmental NGOs and disentangling the contributions to the contamination of distinct waterways made by residential, industrial, and agricultural sewage.

Canal residents strategically seek different water sources based on their perception of quality, cost, access, logistics, and urgency tradeoffs. They clearly seek to minimize harms from contaminated water, despite limited information about scientifically documentable pollution rates. They use water from seeping groundwater pipes [preferentially from *lloraderos*] for self-cleaning and washing possessions and seek packaged or tap water for injecting and consumption. Unwanted contact with polluted river water occurs largely during police raids fleeing through the foul river canal or into wet storm drains. Activities of daily life with limited resources also sometimes inadvertently expose their wounds and skin to harmful water, promoting chronic skin and soft tissue infections (abscesses).

In this context, the existence of contaminated water sources, and routinized exposure to it in the presence of physical insecurity, economic scarcity, and social ostracism, must be understood and highlighted from an *environmental structural violence* framework. These exposures are the result of intersecting structural forces that can be identified and remediated by public health infrastructural improvement. Ultimately, however, such high levels of contamination in the Tijuana River are also a symptom of long-term US policies of economic imperialism in Mexico (Bandy, 2000; Mumme, 2007) and the stark global South/North economic inequality at the highly populated Baja California/California border. Neoliberal policies accelerated by trade agreements, have converted Tijuana into a major manufacturing hub for US corporations. Specific neoliberal and prohibitionist US-Mexico border and migration policies rapidly increased multinational corporation assembly plants and industrial agricultural processing facilities capitalizing on wage disparities at the border which was open for capital but closed for laborers. Tax exemption subsidies to corporations weakened capacity for environmental protection regulations and partially privatized wastewater management encouraging infrastructurally corrupt dumping practices (Bandy, 2000; Ganster, 2005; Gladstone et al., 2021; Mumme, 2007; Villaverde, 2012; Williams and Homedes, 2001). For at least half a century, factories have routinely discharged pollutants and untreated or minimally treated sewage into the Tijuana River with no monitoring and enforcement, escaping legal consequences (Gladstone et al., 2021; Kopinak, 2002; San Diego Regional Water Quality Control Board, 2020). Factory and residential waste routinely contaminate city waterways and drive negative health outcomes among the most vulnerable local residents stranded in the Tijuana River canal zone (Bandy, 2000). An environmental infrastructure lens in public health, consequently, is paramount to identifying the *structural violence* enacted by forcible relegation to this liminal urban public space along the US-Mexico border wall.

The vectors of *structural violence* operating here also interface with class- and race-based power relations and problematic sovereignties on the US-Mexico borderland. As multiple scholars have argued, deportation is a racialized and racializing project disproportionally targeting people of color (De Genova, 2002; Golash-Boza and Hondagneu-Sotelo, 2013). Deportees and the unhoused people are a hyper-visible stigmatized underclass in Tijuana (Contreras Velasco, 2016; Pinillos, 2020). Physical punishment and incarceration are the primary Mexican state response to this community’s objective vulnerabilities. The logics driving poor health outcomes are so imbued in the social fabric that they become the local ‘common-sense’ among front-line police. As has been found in other contexts (Herring, 2019; Herring et al., 2020), the opening fieldnote exposes how visible markers of homelessness are criminalized in the Tijuana River canal. Our research team calmly took water samples and assisted in the distributed harm reduction supplies even as canal inhabitants were violently rounded up and arrested, merely for the crime of being in this same public space. Our clothing, vehicles, and appearance protected us from victimization during these routine police “cleaning” of homeless deportees and internally displaced PWID.

Moreover, The Tijuana River is not a municipal territory, but Federal. Therefore, neither the state of Baja California nor the city of Tijuana has total jurisdiction. Additionally, the Tijuana River is a transboundary river and is ruled by the International Water Agreement between Mexico and the US. Water pollution in the Tijuana River is intrinsically connected and shared by multiple cities and both nations and affects human, animal, and environmental health. Therefore, we argue that solutions are not solely the responsibility of the Tijuana municipality.

## 5. Conclusions

In this mixed-methods analysis, we employ an *environmental structural violence* framework building on the *structural violence* approach to highlight *environmental injustice*. We documented both the social uses and precise chemical toxicities of water in a publicly notorious border-crossing micro-neighborhood. We further analyzed political and economic contextual forces shaping disparities between US/Mexican sovereignties. We also link immediately visible practices of risky contact with contaminated water to: A) deportation by US authorities into a hostile environment with severed social supports, B) inadequate access to WASH services [i.e., drinking water, toilets, showers], C) inadequate sewage treatment and disposal, resulting in waterway pollution, and D) routinized victimization by Mexican law enforcement. These proximal vectors are generated by historical policies and large scale structural forces: 1) Neoliberal free-trade agreements subsidizing private capital investments that foment internal migration without proportional sewage infrastructure investments; 2) prohibitionist US migration laws and executive orders that deport PWID and trap low-income migrants in border cities; 3) the concentration of industrial/agriculture processing and assembly plant factories in border cities that exacerbate contamination of city waterways; 4) the routinization of police abuse of vulnerable populations.

Environmental protection and development of essential public survival infrastructure [WASH, food, shelter, and physical security] is an essential requirement for sustainable economic policy and basic public health and human rights (United Nations, 1992, 2012). Consequently, urgent remediation requires: 1) elimination of tax relief programs for transnational corporations, 2) strengthening of environmental enforcement mechanisms including US sanctions on parent corporations, 3) binational cooperation around comprehensive planning for private and public waste management, and 4) increasing funding for environmental protection and public health monitoring of water toxicity. Pressure from NGOs and the public can foster the repeal of abusive upstream policies that devastate the borderland’s environmental infrastructural sustainability, civil rights and public security of vulnerable populations in *El Bordo*.

Achieving environmental and health justice in this context requires trans-sectoral legislative and community-based interventions to address harms from excessively rapid industrial growth and prohibitionist drug and border control management. Locally, Tijuana and San Diego must develop municipal-level international legislative solutions to ensure canal residents have access to shelter, WASH services, food, and healthcare. Interventions to prevent the systematic brutalization and persecution of vulnerable populations by police are urgently needed. The improvement of urban river water quality requires greater infrastructural investment and binational cooperation. Ultimately, and more ambitiously, the economic neoliberalism and anti-migrant policies of the US that contribute to the phenomenon of injection drug use, and subsequently exacerbate the vulnerability of so many deportees with substance use disorders trapped in the borderland, must be reversed.

## Figures and Tables

**Table 1:** Survey participant characteristics

Variable	n	Value

Age (years)a	84	41.0 (8.9)
Educational attainment (years)a	81	8.0 (2.8)
Male (%)	78	91.8
Female (%)	7	8.2
Indigenous (%)	1	1.2
Place of birth		
Baja California (%)	36	42.4
Southern Mexican States (%)	28	32.9
Northern Mexican States (%)	17	20.0
US (%)	2	2.4
Not answered (%)	2	2.4
Living in the US		
Never lived in the US (%)	33	38.8
Have been lived in the US (%)	52	61.2
Time living in the US (years)b	52	13.0 (0.2–47)
Have been deported from the US (%)	48	56.5
Time since deportation (years)a	34	10.4 (7.4)
Time living/entering *El Bordo* (years)b	82	3.3 (0.003–45)
Household/homelessness status		
Lives in *El Bordo* (%)	43	50.6
Sleeps on the street (%)	6	7.4
Lives in a room, house or apartment (%)	6	7.4
Not defined (%)	6	7.4
Not answered (%)	24	29.6
Employment		
Formal employment (%)	12	14.1
Trade/craft (%)	15	17.6
Informal job/unemployed (%)	58	68.2
Intravenous drug use		
Heroine (%)	13	15.3
Heroine and Methamphetamine (%)	66	77.6
None (%)	6	7.1
Tijuana River canal/lloraderos water use/contact		
Never in last week (%)	25	27.5
At least 1 in last week (%)	66	72.5
1–3 in last week (%)	25	27.5
4–7 in last week (%)	15	16.5
8+ in last week (%)	26	28.6

Source: Self developed table from 85 participants in 2019.

a-mean, b-median.

Southern Mexican States included Michoacan, Jalisco, Guerrero, CDMX, Colima, Edo Mex, Nayarit, San Luis Potosi, and Veracruz. Northern Mexican States included Sonora, Chihuahua, Durango, Sinaloa, and Zacatecas.

The US included California and Texas.

**Table 2:** Tijuana River and/or *lloradero* water use and contact in the last week

Type of use or contact	n	Never (n)	Some days (n)	All week (n)	Use or contact (%)

Drink	95	94	0	1	1.1
Brush teeth	95	88	2	5	7.4
Rinse mouth	95	88	2	5	7.4
Wash hands	95	45	29	21	52.6
Rinse hands	95	35	38	22	63.2
Wash face	94	61	18	15	35.1
Rinse eyes/ears	94	61	21	12	35.1
Rinse face	94	56	25	13	40.4
Bath	95	67	19	9	29.5
Clean wounds	95	77	13	5	18.9
Rinse wounds	94	73	14	7	22.3
Rinse feet	95	46	36	13	51.6
Prepare drug for injection	93	90	2	1	3.2
Cook food	95	86	5	4	9.5
Wash dishes	95	67	16	12	29.5
Laundry	95	59	24	12	37.9

Source: Self developed table from 97 applied questionnaires in 2019.
